# An integrative model with HLA‐DR, CD64, and PD‐1 for the diagnostic and prognostic evaluation of sepsis

**DOI:** 10.1002/iid3.1138

**Published:** 2024-01-10

**Authors:** Guosheng Chen, Huimin Chong, Peng Zhang, Dalin Wen, Juan Du, Chu Gao, Shi Zeng, Ling Zeng, Jin Deng, Kejun Zhang, Anqiang Zhang

**Affiliations:** ^1^ State Key Laboratory of Trauma, Burns and Combined Injury, Institute of Surgery Research, Daping Hospital Army Medical University (Third Military Medical University) Chongqing China; ^2^ Department of Emergency The Affiliated Hospital of Guizhou Medical University Guiyang China; ^3^ Yubei District Hospital of TCM Chongqing China; ^4^ Department of Neurosurgery The People's Hospital of Chongqing Banan District Chongqing China

**Keywords:** CD64, HLA‐DR, PD‐1, predict, sepsis

## Abstract

**Background:**

Sepsis is a life‐threatening organ dysfunction caused by a dysregulated host response to infection and progressive immunosuppression with high mortality. HLA‐DR, CD64, and PD‐1 were assumed to be useful biomarkers for sepsis prediction. However, the ability of a combination of these biomarkers has not been clarified.

**Methods:**

An observational case‐control study was conducted that included 30 sepsis patients, 30 critically ill patients without sepsis admitted to the intensive care unit (ICU), and 32 healthy individuals. The levels of HLA‐DR, CD64, and PD‐1 expression in peripheral blood immune cells and subsets was assayed on Days 1, 3, and 5, and the clinical information of patients was collected. We compared these biomarkers between groups and evaluated the predictive validity of single and combined biomarkers on sepsis mortality.

**Results:**

The results indicate that PD‐1 expression on CD4^−^CD8^−^T (PD‐1^+^CD4^−^CD8^−^T) (19.19% ± 10.78% vs. 9.88% ± 1.79%, *p* = .004) cells and neutrophil CD64 index (nCD64 index) (9.15 ± 5.46 vs. 5.33 ± 2.34, *p* = .001) of sepsis patients were significantly increased, and HLA‐DR expression on monocytes (mHLA‐DR^+^) was significantly reduced (13.26% ± 8.06% vs. 30.17% ± 21.42%, *p* = 2.54 × 10^−4^) compared with nonsepsis critically ill patients on the first day. Importantly, the expression of PD‐1^+^CD4^−^CD8^−^T (OR = 0.622, 95% CI = 0.423–0.916, *p* = .016) and mHLA‐DR^+^ (OR = 1.146, 95% CI = 1.014–1.295, *p* = .029) were significantly associated with sepsis mortality. For sepsis diagnosis, the mHLA‐DR^+^, PD‐1^+^CD4^−^CD8^−^T, and nCD64 index showed the moderate individual performance, and combinations of the three biomarkers achieved greater diagnostic value (AUC = 0.899, 95% CI = 0.792–0.962). When adding PCT into the combined model, the AUC increased to 0.936 (95% CI = 0.840–0.983). For sepsis mortality, combinations of PD‐1^+^CD4^−^CD8^−^T and mHLA‐DR^+^, have a good ability to predict the prognosis of sepsis patients, with an AUC = 0.921 (95% CI = 0.762–0.987).

**Conclusion:**

These findings indicate that the combinations of HLA‐DR, CD64, and PD‐1 outperformed each of the single indicator in diagnosis and predicting prognosis of sepsis.

## Highlights

The present results have shown that three biomarkers of immunosuppression may serve as diagnostic or prognostic markers for septic patients. This was based on the following findings: (1) the percentage of mHLA‐DR+ and PD‐1 + CD4^−^CD8^−^ T cells and the neutrophil CD64 index were significantly different between patients with and without sepsis; furthermore, the percentage of mHLA‐DR+ and PD‐1 + CD4‐CD8‐ T cells was also significantly different between septic patients with and without death in the ICU; (2) there was a significant gain in discriminative power of diagnostic or prognostic sepsis markers when the linear combination that yielded the highest AUC was employed. mHLA‐DR+ and PD‐1 + CD4‐CD8‐T together provide good predictive ability for ICU mortality among subjects with sepsis. The performance of mHLA‐DR+ and PD‐1 + CD4‐CD8‐T in this cohort rivals established clinical‐ and biomarker‐based tools for mortality prediction in sepsis.

## 1 INTRODUCTION

Sepsis represents a life‐threatening organ dysfunction triggered by a dysregulated host response to infection.^1^ Despite improving knowledge of the pathophysiologic process and therapeutic innovations, sepsis has still high morbidity and mortality among hospitalized critically ill patients.^2^ There were nearly 50 million cases of sepsis worldwide, with a mortality rate of 22.5% and accounting for 20% of all global deaths in 2020.^3^ Emerged evidence has indicated that immunosuppression is now recognized as one of the major causes of septic death.^4^ Early monitoring and evaluation of the changes in immune function, timely diagnosis and risk stratification are beneficial to take timely measures to improve sepsis prognosis.^5^ According to Sepsis‐3.0, sepsis was defined as the presence of an infection and organ dysfunction(s) represented by two or more sequential organ failure assessment (SOFA) points. However, there are many challenges in sepsis diagnosis and prognostic evaluation. The source of infection was determined based on the cultivation of pathogenic microorganisms, which, as a gold standard diagnostic method, requires time to confirm, and some other host‐reponse biomarkers, such as procalcitonin (PCT) and C‐reactive protein (CRP), which are used as screening tools, are not sufficiently specific.^6^ Thus, there is an urgent need for a biomarker (or combination of several biomarkers) tool in the diagnosis, risk stratification, prognostication, and treatment management, including administering antimicrobials.

Following the recognition of endogenous danger signals and pathogenic microorganisms, epithelial, endothelial, and immune cells orchestrate a series of inflammatory response.^2^ Simultaneously, immunosuppression initiates in the early phases of sepsis and culminates in the persistent inflammation that contributes to the risk of opportunistic or secondary infection.^7^ More two hundred biomarkers have been proposed over the last few decades, but only a few are potentially useful in clinical practice, such as CRP and PCT.^8^, ^9^ It is necessary to explore more promising biomarkers. The hyperstimulation of immune cells may guide the correct diagnosis and treatment of sepsis. Immune cell surface biomarkers, including HLA‐DR, CD64, and PD‐1, are promising biomarkers for sepsis prediction and evaluation.^6^ HLA‐DR was first proposed as a marker to judge immune paralysis in sepsis patients in 1991.^10^ The persistent decreased expression of mHLA‐DR is a characteristic of sepsis‐related immunosuppression.^11^ The expression of HLA‐DR on monocytes indicates their dysfunction and has been associated with an elevated risk of infections and death in critically ill patients.^12^ In the initial phases of sepsis, the level of CD64 expression increased, which was related to disease severity and 28‐day mortality.^13^ Another checkpoint inhibitor molecule, PD‐1, is increased expressed in septic patients compared to patients with noninfectious inflammation and healthy controls.^14^ However, the single parameter was not robust enough to be used clinically in the field of sepsis.

Therefore, The levels of HLA‐DR, CD64, and PD‐1 expression in immune cell, as well as the percentage of positive cells, were investigated in this study. Based on the results obtained from clinical samples, a panel of combined these biomarkers was constructed and demonstrated excellent performance, which was more accurate than the use of each single biomarker alone.

## 2 METHODS

### 2.1 Study population

This was a prospective observational study performed in the intensive care unit (ICU) of Daping Hospital from February 2021 to February 2022 by enrolling consecutive patients with critically ill patients. The inclusion criteria of critically ill patients were age between 18 and 70 years old. The diagnosis of sepsis was based on the Sepsis‐3 criteria.^15^ The exclusion criteria were patients with autoimmune or hypersensitivity diseases, tumors, or immunodeficiency. Patients with certain infectious diseases, such as HIV, hepatitis B, and syphilis, pregnant women and lactation patients were also withdrawn from the study. Age‐ and gender‐matched healthy volunteers were included in parallel with critically ill patients. All patients were evaluated in the ICU within 24 h after admission. Patients were standardized treated according to the Surviving Sepsis Campaign recommendations.^1^ The study was approved by the Institutional Ethics Review Board of Daping Hospital. All patients provided informed consent from thenselves or their relatives. The National Clinical Trial number is NCT01713205, which was registered on October 22, 2012.

### 2.2 Clinical information collection

Data of patients, including demographic data, existing clinical status, site of infection, disease severity, and organ function, were collected through the clinical patients‐information system. We calculated acute physiology and chronic health evaluation II (APACHE II) score^16^ and SOFA score^17^ to assess the disease severity. Patients were followed up during the ICU period after enrollment. Peripheral venous blood samples were collected from patients on Days 1, 3, and 5 after admission using EDTA anticoagulant.

### 2.3 Flow cytometry analysis

After blood collection, the levels of HLA‐DR, CD64, and PD‐1 expression in peripheral blood samples were detected by flow cytometry (Detailed flow protocols were on Supplement Methods). All anti‐human fluorochromes were from Beckman Coulter Inc., except for CD64 antibodies (BD Pharmingen). Staining was performed with monoclonal antibodies following the manufacturer's instructions.

Cell staining was performed with the following antibodies: Krome Orange‐labeled anti‐CD45, PE. Cy7‐labeled anti‐CD14, APC‐Alexa Fluor 750‐labeled anti‐CD3, PE. Cy7‐labeled anti‐CD4, APC‐Alexa Fluor 700‐labeled Anti‐CD8, Pacific Blue‐labeled Anti‐HLA‐DR, PerCP/cyanine5.5‐labeled anti‐CD279 (PD‐1), and PerCP/Cy5.5‐labeled anti‐CD64. The samples were detected using a Navios multichannel flow cytometer (Beckman Coulter Inc. Brea) and analyzed on the Kaluza Analysis Software, as well as and the Cytobank platform.^18^ All cells were gated by forward scattering (FSC) and lateral scattering. A minimum of 20000 events were analyzed for each sample. In addition, fluorescence minus one control was carried to define positive threshold for HLA‐DR and PD‐1 (Figure S1). The ratio of the mean fluorescence intensity from neutrophils and lymphocytes was calculated as the neutrophil CD64 (nCD64) index.

### 2.4 Statistical analysis

Categorical and continuous variables are expressed as number (proportion) and mean ± standard deviation as appropriate. Comparisons of various individuals were performed with chi‐square tests (for categorical data), unpaired *t*‐test (for normally distributed data), or Mann‐Whitney U test (for nonnormally distributed data). The correlation between variabless was evaluated using the Spearman rank correlation test. A binary logistic regression model was used for the combination analysis of multiple indicators. The discriminate value of each indicator was appraised using the receiver operating characteristic (ROC) curve. Sensitivity and specificity were calculated using a 95% confidence interval (CI). Cox regression analysis was used to analyze the survival time of sepsis patients. SPSS v22.0 (IBM) and GraphPad Prism v8.0 (GraphPad Software) were used for processing data and graphics. For all tests, a two‐sided *p* < .05 was considered to be significant.

## 3 RESULTS

### 3.1 Characteristics of included patients

According to the predetermined criteria, 60 adult critically ill patients, including 30 sepsis patients and 30 nonsepsis patients, and 32 healthy volunteers were enrolled. The main characteristics of the patients are outlined in Table 1. The mean age of sepsis patients was 51.83 years (SD = 11.69), and 40% were female, which was similar to nonsepsis patients. The clinical scoring systems APACHE II and SOFA did not significantly differ between the groups (*p* > .05). Compared with critically ill patients without sepsis, sepsis patients had higher PCT (28.11 ± 32.78 vs. 3.01 ± 5.77, *p* = 1.95 × 10^−4^), CRP (153.83 ± 78.66 vs. 74.05 ± 65.31, *p* = 4.19 × 10^−4^) and creatinine (154.95 ± 125.86 vs. 84.22 ± 98.58, *p* = .025) levels. Among sepsis patients, most patients had a respiratory tract infection (53.33%), followed by urinary tract (43.33%), primary bloodstream (30.00%), and abdominal (26.67%) infections. Gram‐negative infections accounted for approximately 30.00%, gram‐positive infections accounted for 6.67%, and mixed gram‐negative/gram‐positive infections accounted for 50.00% of sepsis cases. Twenty‐three patients had septic shock, and 21 had multiple organ failure. The 9 (30.00%) sepsis patients died during the ICU period.

**Table 1:** Baseline characteristics of the included patients.

Parameters	Critically ill patients		*p*	Sepsis patients		*p*
	Sepsis (*n* = 30)	Nonsepsis (*n* = 30)	value	Nonsurvivors (*n* = 9)	Survivors (*n* = 21)	value
**Demographic characteristics**						
Female, *n* (%)	40.00	30.00	.591	55.56	28.57	.171
Age (years)	51.83 ± 11.69	56.93 ± 9.82	.072	56.43 ± 10.49	58.11 ± 8.51	.675
**Co‐morbidities (*n*)**						
Respiratory diseases	5	0		2	3	
Cardiovascular system diseases	3	3		1	1	
Trauma	5	11		1	5	
Digestive system diseases	12	8		5	7	
Diseases of urinary system	5	2		0	5	
Stroke	0	9		0	0	
**Severity of illness**						
APACHE II score	17.37 ± 5.50	17.53 ± 4.64	.899	21.00 ± 5.07	19.25 ± 2.43	.015
SOFA score	6.77 ± 3.92	6.27 ± 3.79	.617	8.22 ± 3.80	6.14 ± 3.89	.188
**Vital signs**						
T (°C)	38.11 ± 0.99	37.86 ± 0.85	.294	38.01 ± 0.94	38.15 ± 1.03	.728
RR (bpm)	17.86 ± 3.20	20.03 ± 3.76	.628	21.78 ± 10.34	20.24 ± 4.17	.560
Heart rate (bpm)	107.63 ± 21.49	103.70 ± 19.82	.464	112.78 ± 27.78	105.43 ± 18.55	.400
MAP (mmHg)	88.43 ± 15.06	91.62 ± 11.97	.368	87.92 ± 16.87	88.65 ± 14.66	.906
**Laboratory findings**						
WBC (×10^9^/L)	15.98 ± 10.17	10.8 ± 4.95	.010	13.25 ± 8.46	17.15 ± 10.79	.344
Neu (%)	87.21 ± 10.50	83.31 ± 11.78	.008	87.92 ± 8.97	86.90 ± 11.29	.811
CRP (mg/L)	153.83 ± 74.80	79.88 ± 62.91	1.12×10^−4^	156.09 ± 84.12	152.86 ± 72.66	.916
PCT (ng/ml)	33.41 ± 38.52	4.62 ± 8.61	3.56×10^−4^	21.09 ± 27.04	38.69 ± 41.97	.184
Lac (mmol/L)	3.00 ± 3.60	1.86 ± 1.35	.109	4.26 ± 5.23	2.44 ± 2.55	.214
Creatinine (μmol/L)	161.84 ± 132.19	97.00 ± 107.33	.042	101.03 ± 0.50	187.90 ± 141.54	.050
TBIL (μmol/L)	37.02 ± 50.11	38.01 ± 59.37	.944	70.02 ± 82.01	22.87 ± 15.80	.124
ALT (U/L)	173.88 ± 468.99	74.40 ± 135.53	.269	57.27 ± 40.26	223.86 ± 556.36	.382
AST (U/L)	409.25 ± 1194.70	97.00 ± 197.48	.168	107.03 ± 80.56	538.78 ± 1417.14	.374
**Source of infection (*n*)**						
Lungs	16			5	11	
Abdomen	8			3	5	
Bloodstream	9			3	6	
Urinary system	13			5	8	
Other	5			1	3	
**Culture positive, *n* (%)**	26 (86.67%)			8 (88.89%)	18 (85.71%)	
**Organism (*n*)**						
Gram‐positive	2			1	1	
Gram‐negative	9			4	5	
Mixed	15			3	12	
**Hospital length of stay (days)**	27.63 ± 33.65	26.17 ± 19.38	.837	9.89 ± 7.46	35.24 ± 37.64	.057
**ICU length of stay (days)**	15.53 ± 11.58	12.07 ± 0.09	.202	9.67 ± 7.55	18.05 ± 12.23	.068
**ICU mortality *n* (%)**	9 (30.00%)	7 (23.33%)	.559	—	—	

Abbreviations: ALT, alanine aminotransferase; APACHE II, Acute Physiology and Chronic Health Evaluation; AST, aspartate transaminase; CRP, C‐reactive protein; GCS, glasgow coma scale; Lac, lactate; MAP, mean arterial pressure; Neu, Neutrophils; PCT, procalcitonin; RR, respiratory rate; SOFA, sepsis‐related organ failure assessment; T, temperature; TBIL, total bilirubin; WBC, white blood cell.

### 3.2 HLA‐DR, CD64, and PD‐1 expression kinetics

Compared with healthy controls, the percentages of circulating PD‐1^+^/CD3 + T cells, PD‐1^+^/CD4 + T cells, PD‐1/CD8 + T cells, PD‐1 + /CD4 + CD8 + T cells, PD‐1^+^/CD4‐CD8‐T cells, PD‐1^+^/Treg cells, and lymphocytes expressing PD‐1 were significantly higher in septic and nonseptic patients on ICU admission (*p* < .05). Interestingly, only the percentages of PD‐1 + /CD4‐CD8‐T cells were obviously different between septic patients and nonseptic patients (*p* = .004), which was also significantly higher in the nonsurvivors than the survivors of sepsis (*p* = .013). Similar changes were also observed on Days 3 and 5 after ICU admission (*p* < .05).

mHLA‐DR expression decreased in septic or nonseptic patients from Day 1 to 5 compared to healthy controls. Septic patients showed a significantly lower percentage of mHLA‐DR than nonseptic patients over time (*p* < .05). Similarly, nonsurvivors had a significantly greater reduction in mHLA‐DR+ compared to survivors at Day 1 (*p* = .007). The change in mHLA‐DR+ was not statistically significant from Day 3 to 5 (*p* > .05).

**Figure 1:**
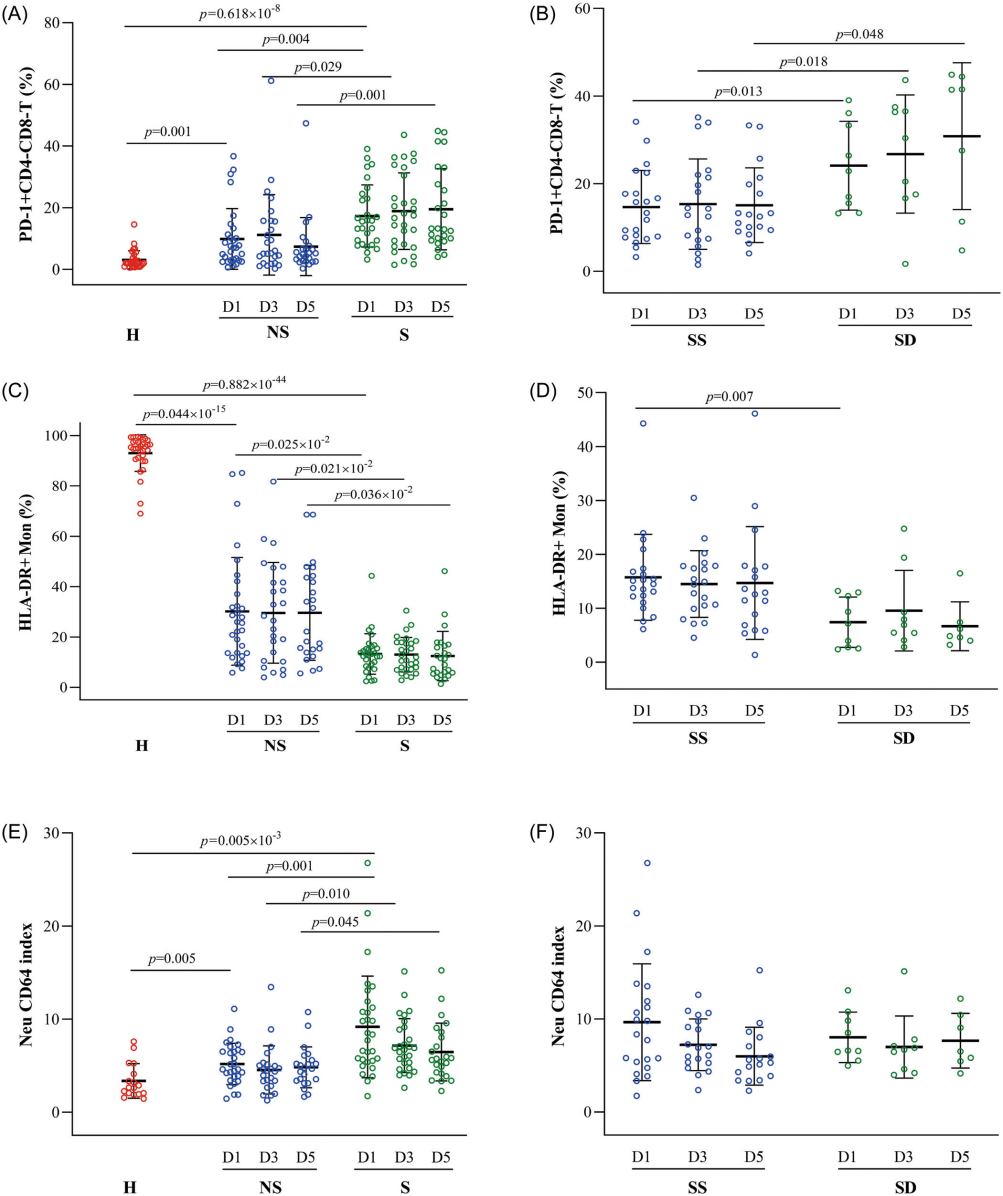
(A–F) Alterations in PD‐1, HLA‐DR, and CD64 expression in the peripheral blood of patients in each group. (A), (C), (E) Dot plots show the percentage of PD‐1 + CD4‐CD48‐T cells and HLA‐DR+ monocytes, the CD64 index in sepsis (S, *n* = 30) and nonsepsis patients (NS, *n* = 30) on Days 1, 3, and 5 of admission, and healthy volunteers (H, *n* = 32). (B), (D), (F) Dot plots show the comparison of three markers between septic survivors (SS) and nonsurvivors (SD) on Days 1, 3, and 5 of admission. D1, Day‐1; D3, Day‐3; D7, Day‐7.

Critically ill patients had a significantly higher nCD64 index than healthy controls (*p* < .05), which was higher in septic patients than in nonseptic patients over time (*p* = .001). However, there were no differences in the nCD64 index between survivors and nonsurvivors at any time point (*p* > .05) (Figure 1, Table 2, and Table S1).

**Table 2:** Comparison of immune cell analysis.

Parameters	Healthy volunteers (*n* = 32)	Critically ill patients		*p*	Sepsis patients		*p*
		Nonsepsis (*n* = 30)	Sepsis (*n* = 30)	value	Survivors (*n* = 21)	Dead (*n* = 9)	value
WBC (×10^9^/L)	5.43 ± 1.32	10.8 ± 4.95	15.98 ± 10.17	.010	17.15 ± 10.79	13.25 ± 8.46	.344
Neu (×10^9^/L)	3.10 ± 0.96	9.07 ± 4.83	14.55 ± 9.65	.008	15.67 ± 10.38	11.93 ± 7.57	.340
Mon (×10^9^/L)	0.29 ± 0.11	0.51 ± 0.30	0.53 ± 0.37	.830	0.57 ± 0.36	0.42 ± 0.39	.327
Lym (×10^9^/L)	1.88 ± 0.43	0.82 ± 0.45	0.75 ± 0.45	.830	0.83 ± 0.46	0.57 ± 0.41	.149
CD3 + T(%)	70.22 ± 7.27	32.29 ± 13.34	45.54 ± 15.02	.001	55.82 ± 16.44	50.91 ± 10.67	.419
CD4 + CD8‐T(%)	35.42 ± 0.61	18.64 ± 1.78	27.49 ± 1.90	.438	32.78 ± 1.78	32.68 ± 1.73	.284
CD4‐CD8 + T(%)	28.92 ± 0.61	11.32 ± 1.62	15.52 ± 1.85	.759	19.99 ± 1.79	15.28 ± 1.61	.243
CD4 + CD8 + T(%)	0.51 ± 0.04	0.34 ± 0.15	0.58 ± 0.21	.530	0.57 ± 0.13	0.95 ± 0.24	.311
CD4‐CD8‐T(%)	5.39 ± 0.33	1.98 ± 0.55	1.95 ± 0.41	.046	2.48 ± 0.49	2.00 ± 0.25	.653
Treg (%)	2.38 ± 0.16	4.27 ± 0.77	4.69 ± 0.37	.745	4.47 ± 0.35	5.24 ± 0.33	.644
**HLA‐DR expression**							
CD14 + HLA‐DR + (%)	92.83 ± 7.26	30.17 ± 21.42	13.26 ± 8.06	2.54×10^−4^	15.76 ± 7.98	7.42 ± 4.66	.007
CD3 + CD4 + HLA‐DR + (%)	19.67 ± 5.76	16.07 ± 8.02	11.45 ± 6.06	.015	12.16 ± 6.62	9.79 ± 4.39	.336
CD3 + CD8 + HLA‐DR + (%)	28.02 ± 6.37	30.95 ± 10.89	27.41 ± 16.23	.326	26.20 ± 15.44	30.22 ± 18.60	.544
**CD64 expression**							
mCD64 Index	13.92 ± 2.20	19.84 ± 6.20	25.74 ± 11.31	.016	27.45 ± 12.55	21.75 ± 6.67	.212
nCD64 Index	3.34 ± 1.86	5.33 ± 2.34	9.15 ± 5.46	.001	9.64 ± 6.29	8.02 ± 2.72	.467
**PD‐1 expression**							
Neu PD‐1 + (%)	62.06 ± 27.27	43.78 ± 30.89	34.05 ± 23.98	.178	32.32 ± 17.82	38.08 ± 35.55	.556
Mon PD‐1 + (%)	13.46 ± 11.50	10.26 ± 7.46	16.16 ± 12.01	.053	18.12 ± 13.19	11.59 ± 7.39	.176
Lym PD‐1 + (%)	4.10 ± 2.41	10.76 ± 6.97	12.33 ± 4.52	.304	11.96 ± 4.26	13.21 ± 5.22	.497
CD3 + PD‐1 + (%)	5.78 ± 4.11	14.50 ± 8.92	18.93 ± 6.72	.034	17.43 ± 5.62	22.44 ± 8.07	.060
PD‐1 + CD4 + CD8‐T(%)	7.07 ± 5.60	16.65 ± 9.43	20.87 ± 8.21	.070	19.42 ± 7.76	24.25 ± 8.70	.142
PD‐1 + CD4‐CD8 + T(%)	4.62 ± 3.63	13.74 ± 10.61	15.35 ± 6.72	.452	15.05 ± 6.50	16.05 ± 7.58	.717
PD‐1 + CD4 + CD8 + T(%)	10.84 ± 11.41	30.67 ± 21.27	39.10 ± 18.37	.960	37.51 ± 19.04	44.57 ± 21.47	.377
PD‐1 + CD4‐CD8‐ T(%)	3.19 ± 2.99	9.88 ± 1.79	19.19 ± 10.78	.004	14.67 ± 8.36	24.10 ± 10.12	.013
Treg PD‐1 + (%)	7.42 ± 8.36	21.11 ± 12.94	27.72 ± 14.28	.065	25.03 ± 12.84	34.02 ± 16.21	.115
**CRP (mg/L)**	—	79.88 ± 62.91	153.83 ± 74.80	1.12×10^−4^	152.86 ± 72.66	156.09 ± 84.12	.916
**PCT (ng/ml)**	—	4.62 ± 8.61	33.41 ± 38.52	3.56×10^−4^	38.69 ± 41.97	21.09 ± 27.04	.184
**Lac (mmol/L)**	—	1.86 ± 1.35	3.00 ± 3.60	.109	2.44 ± 2.55	4.26 ± 5.23	.214

*Note*: Neutrophil CD64 (nCD64) index was the ratio of the mean fluorescence intensity from neutrophils and lymphocytes. Monocyte CD64 (mCD64) index was the ratio of the mean fluorescence intensity from monocytes and lymphocytes.

Abbreviations: HLAHLA‐DR,‐DR, human leukocyte antigen DR; Lym, lymphocytes; Mon, Monocytes; PD‐1, programmed cell death‐1.

**Table 3:** Logistic regression analyses of these biomarkers for differentiating septic and nonseptic patients.

Variables	Crude		Adjusted^a^	
	OR (95% CI)	*p* value	OR (95% CI)	*p* value
mHLA‐DR^+^(%)	0.898 (0.838–0.962)	.002	0.896 (0.835–0.961)	.002
nCD64 index	1.406 (1.125–1.755)	.003	1.421 (1.118–1.807)	.004
PD‐1^+^CD4^−^CD8^‐^T(%)	1.118 (1.039–1.203)	.003	1.089 (1.023–1.159)	.007
PCT (ng/mL)	1.067 (1.015–1.122)	.011	1.088 (1.023–1.158)	.008

^a^ Adjusted for gender, age, SOFA score, and APACHE II score.

### 3.3 Clinical performance of these biomarkers in the diagnosis of sepsis

For discrimination of sepsis within critically ill patients, the ability of PD‐1 + CD4‐CD8‐T cell, mHLA‐DR, and nCD64 index on the first day were evaluated using univariate logistic regression. The three biomarkers were statistically significant in diagnosing sepsis from critically ill patients and were still associated with sepsis adjusted for sex, age, SOFA score, and APACHE II score (*p* < .05) (Table 3). The areas under the ROC curves (AUCs) differed among these three biomarkers, with PD‐1 + CD4‐CD8‐T at 0.776 (0.648–0.875), mHLA‐DR at 0.786 (0.661–0.881), and the nCD64 index at 0.752 (0.623–0.855). The diagnostic ability of the combination of PD‐1, CD64, and HLA‐DR was evaluated by multinomial logistic regression. For the analysis, the AUC for the combined panel, including PD‐1 + CD4‐CD8‐T, mHLA‐DR+, and nCD64 index, was 0.899 (0.792–0.962), and the combined panel and PCT achieved a higher AUC of 0.936 (0.840–0.983), while PCT alone achieved an AUC of 0.851 (0.736–0.930), indicating an excellent diagnostic ability and outperforming any single parameter (Table 4).

**Table 4:** Comparison of clinical performance of biomarkers in diagnosing sepsis.

Variables	AUC (95% CI)	Optimal	Sensitivity	Specificity	Youden	PPV	NPV
		Cut‐off value	(%)	(%)	Index (%)	(%)	(%)
APACHE II score	0.524 (0.391–0.654)	≤18	63.33	50.00	0.13	55.88	57.69
SOFA score	0.527 (0.394–0.658)	>7	46.67	76.67	0.23	66.67	58.98
CRP (mg/L)	0.769 (0.641–0.869)	>76.22	86.21	56.67	0.43	66.55	80.43
PCT (ng/ml)	0.851 (0.736–0.930)	>2.33	86.67	73.33	0.60	76.47	84.62
mHLA‐DR^+^(%)	0.786 (0.661–0.881)	≤17.39	86.67	66.67	0.53	72.23	83.34
nCD64 index	0.752 (0.623–0.855)	>7.76	50.00	93.10	0.43	87.87	65.06
PD‐1^+^CD4^‐^CD8^‐^T(%)	0.776 (0.648–0.875)	>11.33	67.86	76.67	0.45	74.42	70.46
Combined panel	0.899 (0.792–0.962)	>0.58	83.33	86.21	0.70	85.80	83.80
PCT+Combined panel	0.936 (0.840–0.983)	>0.40	90.00	86.21	0.76	86.71	89.61

*Note*: Combined panel, mHLA‐DR^+^ + nCD64 index + PD‐1 + CD4‐CD8‐T.

### 3.4 Predictive performance of these biomarkers for ICU mortality in sepsis patients

The percentages of mHLA‐DR+ and PD‐1 + CD4‐CD8‐T cells on ICU admission were significantly different in the nonsurvivors compared to the survivors using univariate logistic regression and adjusted for sex, age, SOFA score, and APACHE II score. However, no difference was observed in the nCD64 index between survivors and nonsurvivors (Table 5). Furthermore, the AUCs of the positive percentage of mHLA‐DR+ and PD‐1 + CD4‐CD8‐T cells for predicting ICU mortality were 0.868 (0.764–0.999) and 0.759 (0.580–0.939), respectively, which were higher than that of the SOFA score (AUC = 0.661 [0.458–0.865]). The AUC of the combination of the two biomarkers was 0.921 (0.762–0.987) (Table 6).

**Table 5:** Logistic regression analyses of these biomarkers for differentiating survivors and nonsurvivors.

Variable	Crude		Adjusted^a^	
	OR (95% CI)	*p* value	OR (95% CI)	*p* value
PCT(ng/ml)	0.985 (0.961–1.011)	.258	0.976 (0.942–1.011)	.174
mHLA‐DR^+^(%)	0.715 (0.557–0.919)	.009	0.622 (0.423–0.916)	.016
nCD64 index	0.938 (0.792–1.110)	.457	0.880 (0.683–1.133)	.320
PD‐1^+^CD4^−^CD8^‐^T(%)	1.115 (1.013–1.228)	.026	1.146 (1.014–1.295)	.029

^a^ Adjusted for gender, age, SOFA score, and APACHE II score.

**Table 6:** Comparison of clinical performance of biomarkers in predicting sepsis mortality.

Variables	AUC (95% CI)	Optimal	Sensitivity	Specificity	Youden	PPV	NPV
		Cut‐off value	(%)	(%)	index (%)	(%)	(%)
APACHE II score	0.773 (0.584–0.961)	>19	66.67	80.95	0.48	60.00	85.00
SOFA score	0.661 (0.458–0.865)	>3	100.00	38.10	0.38	40.91	100.00
PCT (ng/mL)	0.603 (0.395–0.811)	≤60.01	88.89	38.10	0.27	38.10	88.89
mHLA‐DR^+^(%)	0.868 (0.764–0.999)	≤13.24	100.00	61.90	0.62	52.94	100.00
PD‐1^+^CD4^−^CD8^−^ T(%)	0.759 (0.580–0.939)	>11.76	100.00	47.62	0.48	45.00	100.00
Combined panel	0.921 (0.762–0.987)	>0.38	77.78	95.24	0.73	87.50	90.91

*Note*: Combined panel, mHLA‐DR^+^ + PD‐1^+^CD4^−^CD8^‐^T.

### 3.5 Survival curves

**Figure 2:**
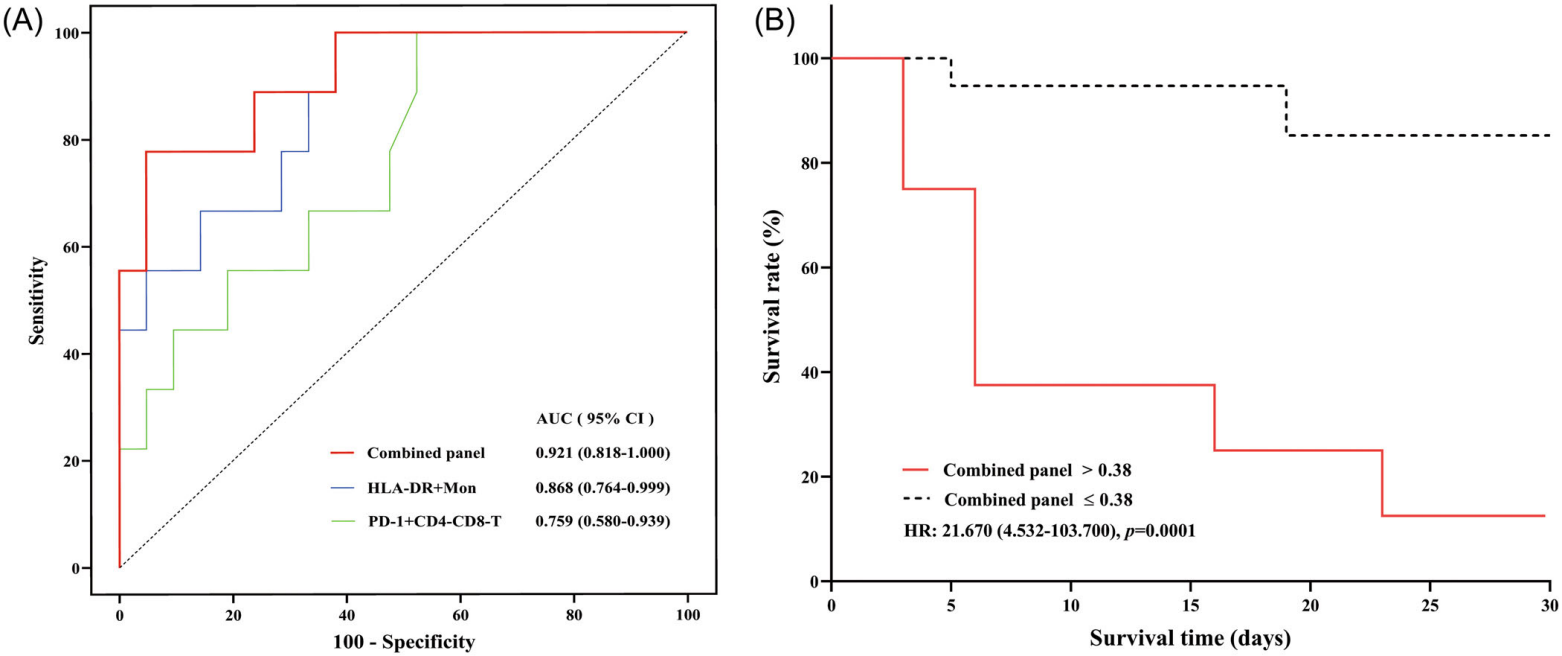
(A), (B) ROC analyses for predicting ICU mortality. (C)‐(H) Cox regression analysis of survival curves. (A) The AUC and 95% CI of mHLA‐DR, PD‐1 + CD4‐CD8‐T, and the combined model constructed by the two indicators. (B) Cox regression analysis survival curves showed that sepsis patients with the predicted probability of the combined model ≥0.38 had higher ICU mortality (HR = 21.67, 95% CI = 4.53–103.70, *p* = .0001) than those with lower levels.

During hospitalization in the ICU, survival of sepsis patients was 70.0%. Cox regression analysis revealed that septic patients with a lower percentage of mHLA‐DR+ than the cutoff value of 13.24% had a higher mortality (hazard ratio [HR] = 5.90, 95% CI = 1.52–22.92, *p* = .01), as compared to those with a higher percentage of mHLA‐DR + . Conversely, there was higher mortality (HR = 4.54, 95% CI = 1.11–18.57), *p* = .03) in septic patients with higher percentage of PD‐1 + CD4‐CD8‐T cells than the cutoff of 11.76%. Additionally, we calculate the predicted probability of combination the percentage of mHLA‐DR+ and PD‐1 + CD4‐CD8‐T cells used the regression equation model. The results showed that the predicted probability of the combined panel with higher than the cutoff of 0.38 was higher ICU mortality (HR = 21.67, 95% CI = 4.53–103.70, *p* = .0001) compared to patients with lower predicted probability (Figure 2).

### 3.6 Correlation analysis of mHLA‐DR, nCD64, and PD‐1 + CD4‐CD8‐T cells with clinical severity

No significant correlations were observed between the percentage of mHLA‐DR+ and APACHE II scores on Day 1 (*p* > .05). However, mHLA‐DR + % was negatively correlated with APACHE II D3 (ρ = −.544, *p* = .004), APACHE II D5 (ρ = −.413, *p* = .040), SOFA score D1 (ρ = −.383, *p* = .044), SOFA score D3 (ρ = −.460, *p* = .016), and SOFA score D5 (ρ = −.511, *p* = .009). PD‐1 + CD4‐CD8‐T% was positively correlated with APACHE II D5 (ρ = .409, *p* = .034). nCD64 index was negatively correlated with APACHE II D1 (ρ = −.454, *p* = .012), APACHE II D3 (ρ = −.458, *p* = .014), and APACHE II D5 (ρ = −.389, *p* = .045).

## 4 DISCUSSION

The present study has indicated that three biomarkers of immunosuppression may serve as diagnostic or prognostic markers for septic patients. The was based on the following findings: (1) the percentage of mHLA‐DR +, PD‐1 + CD4‐CD8‐T cells, and the nCD64 index were significantly different between patients with and without sepsis; furthermore, the proportion of mHLA‐DR+ and PD‐1 + CD4‐CD8‐T cells was also significantly different between septic patients who died in the ICU and those who survived; (2) there was a marked gain in discriminative power of diagnostic or prognostic markers of sepsis when the combination that yielded the highest AUC was employed; and (3) adding PCT has improved the discriminative power of combined panel.

There's growing evidence that the immune system is fundamentally important in the progression of sepsis. The alteration in the function and percent of immune cell subsets have a great impact on the inflammatory response in different stages of sepsis pathogenesis. Sepsis‐induced hyperstimulation of immune cells may provide crucial guide for accurate diagnosis and treatment. Immune cell surface markers, containing HLA‐DR, CD64, and PD‐1, are promising biomarkers for sepsis diagnosis and prognosis.^19^ mHLA‐DR has been confirmed to be a reliable indicator for estimating sepsis‐induced immunesuppression.^20^, ^21^ The percentage of mHLA‐DR+ significantly decreased during sepsis, and the dynamic change in mHLA‐DR+ over time may be a reliable predictor for sepsis mortality.^22^ CD64 is an IgG‐binding receptor expressed by neutrophils, monocytes and lymphocytes in response to cytokines released during bacterial infection.^23^ The nCD64 index has been studied for years as a potential diagnostic biomarker of sepsis with good sensitivity and specificity.^24^, ^25^ PD‐1 (CD279) and its two ligands, PD‐L1 (CD274) and PD‐L2 (CD273) constitute a complex system of negative regulators involved in controlling T‐cell responses in sepsis. In comparison with trauma patients and healthy volunteers, PD‐1 expression is increased on monocytes and CD4 + T cells after sepsis.^26^ As shown in this study, we evaluated HLA‐DR expression in monocytes, PD‐1 expression in T cells and their subsets, and the nCD64 index to diagnose sepsis and its prognosis and found that mHLA‐DR + , PD‐1 + CD4‐CD8‐T cells, and the nCD64 index had the best indicating performance, both individually and in combination. To the best of our current understanding, this is the initial report of the utility of the percentage of PD‐1 + CD4‐CD8‐T cells in predicting mortality associated with sepsis. Furthermore, the performance of three biomarkers in combinations matched or exceeded the ability of clinical practice scoring system (APACHE II and SOFA score) and other, more extensively inflammatory biomarkers (CRP and PCT) used for morbidity and mortality prediction in sepsis. The prediction ability of the combinations is better than that of each single marker. Our findings suggest that mHLA‐DR, PD‐1 of CD4‐CD8‐T cells, and the nCD64 index, when combined with plasma PCT, may prove a valuable tool to predict mortality in sepsis. These results are consistent with previous studies,^27^, ^28^ such as nCD64 expression combined with the SOFA score is a valuable panel for early diagnosis of infection in sepsis, risk stratification and evaluation of prognosis in sepsis patients in the emergency department.^29^ In additon, these findings have potential clinical relevance and biological plausibility. The crucial pathophysiological insight is that leukocyte biomarkers of immunosuppression, such as antigen processing ability (HLA‐DR) and check‐point inhibitors (PD‐1, PD‐L1), were altered even in sepsis patients admitted to the ICU. These markers were present on the vital innate immune cells, containing monocytes and neutrophils, which are the first line of defense to infection.^30^

Several potential limitations are worth highlighting. Firstly, this is a monocentric and relatively small sample preliminary study, and the identification of these biomarker combinations may not be applied elsewhere. Further confirmation is greatly needed. Secondly, due to limited prehospital information, containing the use of antibiotics, steroids, anti‐inflammatory drugs, and catecholamines, and so forth, the findings were not corrected for confounding factors, which need to be considered in future study. Thirdly, the current study only defined ICU mortality as the outcome and did not conduct further follow‐up; thus, it is possibly unable to indicate the long‐term effect of sepsis. Finally, flow cytometry data was still used for study only and not for general clinical practice at present time. Notwithstanding the aforementioned limitations, these finding that, when assayed in subjects who meet sepsis criteria, mHLA‐DR+ and PD‐1 + CD4‐CD8‐T have predictive validity for ICU mortality merits close monitoring. Models developed using mHLA‐DR+ and PD‐1 + CD4‐CD8‐T cells outperformed more established clinical and biomarker‐based tools in this cohort. It is crucial to further characterize the dysregulated immune response that underlies sepsis and its associated mortality. These findings that mHLA‐DR+ and PD‐1 + CD4‐CD8‐T predict mortality risk early in the course of sepsis may contribute to improved models of sepsis‐related immune dysregulation, risk‐stratification, and personalized treatment strategies.

In conclusions, the combination of mHLA‐DR+ and PD‐1 + CD4‐CD8‐T provide better predictive ability for ICU mortality among sepsis subjects, which rivals established clinical‐ and biomarker‐based tools in this cohort. Further research should be directed toward validating mHLA‐DR+ and PD‐1 + CD4‐CD8‐T for sepsis prognosis and assessing these additive value in combination with predictive clinical features.

## AUTHOR CONTRIBUTIONS

**Guosheng Chen**: Data curation; methodology; writing—original draft. **Huimin Chong**: Methodology; supervision; validation. **Peng Zhang**: Resources; software; validation; visualization. **Dalin Wen**: Data curation; investigation; methodology. **Juan Du**: Formal analysis; software; supervision. **Chu Gao**: Validation. **Shi Zeng**: Resources; software. **Ling Zeng**: Writing—review and editing. **Jin Deng**: Funding acquisition; supervision; visualization; writing—review and editing. **Kejun Zhang**: Methodology; resources; supervision; writing—review and editing. **Anqiang Zhang**: Conceptualization; project administration; writing—review and editing.

## CONFLICT OF INTEREST STATEMENT

The authors declare no conflict of interest.

## ETHICS STATEMENT

The study was approved by the Institutional Ethics Review Board of the Daping Hospital (No. AMMU2019137). Informed consent of all patients was obtained from the patients or their relatives.

## Supporting information


**Supplementary Figure 1. Flow dot plots of immune subsets. (A), (B)** Comparison of CD14 + HLA‐DR percentage and PD‐1 + CD4‐CD8‐T percentage in peripheral blood leukocyte flow cytometry density map of healthy volunteers, nonsepsis patients, sepsis survivors and nonsurvivors. Among them, HLA‐DR and PD‐1 used FMO control to determine the range of cells.

Supporting information.

## Data Availability

The datasets presented in this study can be found in online repositories. The names of the repository/repositories and accession number(s) can be found in the article/Supplementary Material. The datasets used and/or analyzed during the current study are available from the corresponding author on reasonable request.
