# Supplementary material for: An integrative model with HLA‐DR, CD64, and PD‐1 for the diagnostic and prognostic evaluation of sepsis
Source: Immun Inflamm Dis. 2024 Jan 10;12(1):e1138. doi: 10.1002/iid3.1138 (PMC10777881; doi:10.1002/iid3.1138)
Supplement: Supplementary file 2 — Supporting information. [file IID3-12-e1138-s001.docx]

**Supplementary Materials**

**Flow protocols**

**Blood collection:** 4ml arterial blood was collected with K2 EDTA anticoagulant tubes (BD Vacutainer®,367230) and processed within 4 hours. **Cell preparation:** the blood collected from each patient was transferred to two 5mL round-bottom FACS tubes for processing, each 300ul, and add 3ml of red blood cell lysis solution (Beckman Coulter Inc) to each tube. Vortex mixing, dissolve at room temperature for 20 minutes, add 500ml of phosphate-buffered saline (PBS), vortex mixing and terminate the lysis. 500g / min, centrifuge in a low-speed centrifuge for 5 minutes, discard the supernatant, add 1ml PBS again, and repeat the above operation. **Staining:** All anti-human fluorochromes were from Beckman Coulter Inc., except for CD64 antibodies (BD Pharmingen, USA). Staining was performed with monoclonal antibodies and homotypic controls according to the manufacturer's recommendations. Antibodies must be titrated in accordance with the manufacturer’s. The addition amount of all antibodies was 5ul. Add Krome Orange-labeled anti-CD45, PE. Cy7-labeled anti-CD14, PerCP/Cy5.5-labeled anti-CD64 and Pacific Blue-labeled Anti-HLA-DR into the first loading tube, and incubate for 20 minutes at room temperature in the dark. Add Krome Orange-labeled anti-CD45, APC-Alexa Fluor 750-labeled anti-CD3, PE. Cy7-labeled anti-CD4, APC-Alexa Fluor 700-labeled Anti-CD8 and PerCP/cyanine5.5-labeled anti-CD279 (PD-1) to the second loading tube and incubate for 20 minutes under the same conditions. Add 1ml of PBS to each loading tube, vortex and mix for washing, 500g / min, centrifuge for 5 minutes, discard the supernatant, and repeat washing once. Add 400ul PBS again and wait for flow cytometry analysis. In addition, each sample must have fluorescence minus

one (FMO) control for HLA-DR and CD279. **Analysis Sample:** the cells were evaluated by a researcher who did not know our clinical data. The samples were processed on a Navios multichannel flow cytometer (Beckman Coulter Inc. Brea, CA, USA). All cells were gated by forward scattering (FSC) and lateral scattering (SSC).

PMT voltages for FSC, SSC and each emission wavelengths must be adjusted in advance, followed by running single-stained samples for the compensation of spectral overlapping of fluorochrome emission. Data analysis was determined for a minimum of 20 000 events for each blood sample. **Data analysis:** analyzed using Kaluza Analysis Software 2.1 and the Cytobank platform.Except for CD64, which is expressed by mean fluorescent intensity (MFI), all other indicators are expressed by the percentage of positive expression. Immune cell subtypes were defined and gated by their characteristic forward and side scatter profiles that represent the size and morphology of the cells, respectively. The selection steps of target cell populations are shown in supplementary Figure 1.

**Supplementary Table 1. Dynamic changes of immunological biomarkers in critically ill patients.**

| **Parameters** | **D1** | | | | | | **D3** | | | | | | **D5** | | | | | |
| --- | --- | --- | --- | --- | --- | --- | --- | --- | --- | --- | --- | --- | --- | --- | --- | --- | --- | --- |
|  | **Critically ill patients** | | ***p*** | **Sepsis patients** | | ***p*** | **Critically ill patients** | | ***p*** | **Sepsis patients** | | ***p*** | **Critically ill patients** | | ***p*** | **Sepsis patients** | | ***p*** |
|  | **S (n=30 )** | **NS (n=30 )** | **value** | **SD (n=9 )** | **SS (n=21 )** | **value** | **S (n=30 )** | **NS (n=30 )** | **value** | **SD (n=9 )** | **SS (n=21 )** | **value** | **S (n=30 )** | **NS (n=30 )** | **value** | **SD (n=9 )** | **SS (n=21 )** | **value** |
| **WBC (×10^9^/L)** | 15.98±10.17 | 10.8±4.95 | 0.010 | 13.25±8.46 | 17.15±10.79 | 0.344 | 15.75±10.43 | 10.48±3.45 | 0.014 | 16.06±12.03 | 15.61±9.97 | 0.916 | 14.51±7.94 | 11.21±4.23 | 0.068 | 16.65±11.32 | 13.72±6.51 | 0.415 |
| Neu (×10^9^/L) | 14.55±9.65 | 9.07±4.83 | 0.008 | 11.93±7.57 | 15.67±10.38 | 0.340 | 13.88±9.34 | 9.11±3.37 | 0.014 | 13.53±9.37 | 14.04±9.57 | 0.895 | 12.03±6.57 | 9.39±3.76 | 0.081 | 12.58±8.12 | 11.83±6.15 | 0.800 |
| Mon (×10^9^/L) | 0.53±0.37 | 0.51±0.30 | 0.830 | 0.42±0.39 | 0.57±0.36 | 0.327 | 0.53±0.42 | 0.57±0.27 | 0.634 | 0.45±0.57 | 0.56±0.34 | 0.548 | 0.81±0.59 | 0.75±0.39 | 0.671 | 0.68±0.73 | 0.85±0.54 | 0.518 |
| Lym (×10^9^/L) | 0.75±0.45 | 0.82±0.45 | 0.830 | 0.57±0.41 | 0.83±0.46 | 0.149 | 0.74±0.46 | 0.78±0.56 | 0.262 | 0.48±0.29 | 0.85±0.48 | 0.042 | 0.90±0.64 | 0.96±0.57 | 0.289 | 1.10±1.02 | 0.83±0.45 | 0.512 |
| CD3+(%) | 45.54±15.02 | 32.29±13.34 | 0.001 | 50.91±10.67 | 55.82±16.44 | 0.419 | 42.26±15.43 | 32.59±14.49 | 0.020 | 50.99±10.67 | 55.82±16.44 | 0.118 | 43.37±17.01 | 30.12±11.67 | 0.003 | 43.76±11.33 | 61.54±16.39 | 0.015 |
| CD4+T(%) | 27.49±1.90 | 18.64±1.78 | 0.438 | 32.68±1.73 | 32.78±1.78 | 0.284 | 63.90±11.67 | 57.55±14.07 | 0.073 | 68.45±12.60 | 61.85±10.94 | 0.163 | 64.95±13.73 | 61.69±13.73 | 0.289 | 68.33±13.14 | 63.63±10.27 | 0.352 |
| CD8+T(%) | 4.69±0.37 | 4.27±0.77 | 0.745 | 5.24±0.33 | 4.47±0.35 | 0.644 | 29.93±10.42 | 34.16±11.32 | 0.043 | 25.14±10.29 | 32.08±10.00 | 0.098 | 30.47±10.82 | 30.12±10.92 | 0.010 | 27.34±12.47 | 31.68±10.25 | 0.380 |
| CD4+CD8+T(%) | 15.52±1.85 | 11.32±1.62 | 0.759 | 15.28±1.61 | 19.99±1.79 | 0.243 | 1.52±1.45 | 1.03±0.71 | 0.110 | 1.86±2.29 | 1.02±0.80 | 0.936 | 1.44±1.70 | 1.56±2.40 | 0.833 | 1.77±1.48 | 1.30±1.80 | 0.546 |
| CD4-CD8-T(%) | 0.58±0.21 | 0.34±0.15 | 0.530 | 0.95±0.24 | 0.57±0.13 | 0.311 | 4.65±3.93 | 7.27±5.38 | 0.043 | 4.85±4.67 | 4.56±4.68 | 0.857 | 3.15±1.60 | 6.63±5.90 | 0.010 | 2.55±1.06 | 3.38±1.73 | 0.252 |
| Treg(%) | 1.95±0.41 | 1.98±0.55 | 0.046 | 2.00±0.25 | 2.48±0.49 | 0.653 | 7.95±3.84 | 7.64±3.53 | 0.756 | 10.36±4.33 | 6.87±3.14 | 0.021 | 8.52±3.32 | 7.50±3.88 | 0.328 | 10.58±2.97 | 7.72±3.17 | 0.070 |
| **HLA-DR expression** |  |  |  |  |  |  |  |  |  |  |  |  |  |  |  |  |  |  |
| CD14+HLA-DR+(%) | 13.26±8.06 | 30.17±21.42 | 2.54×10^-4^ | 7.42±4.66 | 15.76±7.98 | 0.007 | 12.98±6.87 | 29.56±20.03 | 2.14×10^-4^ | 9.57±7.48 | 14.51±6.17 | 0.073 | 12.45±9.80 | 29.61±18.90 | 3.55×10^-4^ | 6.68±4.54 | 14.69±10.46 | 0.065 |
| CD3+CD4+HLA-DR+(%) | 11.45±6.06 | 16.07±8.02 | 0.015 | 9.79±4.39 | 12.16±6.62 | 0.336 | 13.21±7.84 | 18.67±7.50 | 0.011 | 13.02±6.14 | 13.29±8.64 | 0.933 | 13.75±6.61 | 17.82±8.83 | 0.074 | 16.19±3.05 | 12.80±7.42 | 0.119 |
| CD3+CD8+HLA-DR+(%) | 27.41±16.23 | 30.95±10.89 | 0.326 | 30.22±18.60 | 26.20±15.44 | 0.544 | 26.02±12.29 | 31.97±10.47 | 0.060 | 28.14±12.29 | 25.07±12.49 | 0.544 | 25.46±9.90 | 34.66±11.75 | 0.005 | 28.10±9.60 | 24.43±10.08 | 0.418 |
| **CD64 expression** |  |  |  |  |  |  |  |  |  |  |  |  |  |  |  |  |  |  |
| Lym CD64 MFI | 0.53±0.16 | 0.55±0.37 | 0.786 | 0.52±0.12 | 0.54±0.18 | 0.737 | 0.55±0.14 | 0.50±0.96 | 0.219 | 0.53±0.95 | 0.55±0.16 | 0.742 | 0.54±0.97 | 0.52±0.10 | 0.495 | 0.54±0.89 | 0.54±0.10 | 0.945 |
| Mon CD64 MFI | 13.09±6.01 | 9.98±2.88 | 0.014 | 11.13±3.96 | 13.94±6.61 | 0.248 | 10.73±3.11 | 9.48±3.08 | 0.154 | 9.71±2.50 | 11.21±3.32 | 0.243 | 9.73±3.52 | 9.16±3.35 | 0.576 | 8.42±4.18 | 10.28±3.19 | 0.248 |
| Neu CD64 MFI | 4.71±2.92 | 2.91±2.02 | 0.007 | 4.22±1.82 | 4.93±3.30 | 0.550 | 3.91±2.06 | 2.25±1.15 | 0.001 | 3.69±1.80 | 4.02±2.21 | 0.698 | 3.40±1.45 | 2.46±1.08 | 0.016 | 4.17±1.75 | 3.08±1.22 | 0.092 |
| Mon CD64 Index | 25.74±11.31 | 19.84±6.20 | 0.016 | 21.75±6.67 | 27.45±12.55 | 0.212 | 20.38±6.25 | 19.46±7.52 | 0.633 | 18.93±5.69 | 21.07±6.54 | 0.408 | 18.40±6.72 | 17.87±7.01 | 0.795 | 15.95±8.41 | 19.40±5.89 | 0.262 |
| Neu CD64 Index | 9.15±5.46 | 5.33±2.34 | 0.001 | 8.02±2.72 | 9.64±6.29 | 0.467 | 7.13±2.91 | 4.53±2.58 | 0.001 | 6.97±3.35 | 7.21±2.78 | 0.841 | 6.46±3.10 | 4.81±2.19 | 0.042 | 7.65±2.94 | 5.97±3.11 | 0.237 |
| **PD-1 expression** |  |  |  |  |  |  |  |  |  |  |  |  |  |  |  |  |  |  |
| Neu PD-1+(%) | 34.05±23.98 | 43.78±30.89 | 0.178 | 38.08±35.55 | 32.32±17.82 | 0.556 | 34.42±25.08 | 26.13±23.82 | 0.216 | 34.66±25.95 | 34.31±25.37 | 0.973 | 33.58±26.85 | 32.90±28.54 | 0.931 | 26.77±18.36 | 36.22±28.89 | 0.432 |
| Mon PD-1+(%) | 16.16±12.01 | 10.26±7.46 | 0.053 | 11.59±7.39 | 18.12±13.19 | 0.176 | 19.91±17.47 | 10.26±7.46 | 0.010 | 16.46±15.04 | 21.46±18.61 | 0.486 | 15.59±10.36 | 13.57±11.29 | 0.517 | 18.08±12.25 | 14.62±9.75 | 0.465 |
| Lym PD-1+(%) | 12.33±4.52 | 10.76±6.97 | 0.304 | 13.21±5.22 | 11.96±4.26 | 0.497 | 15.07±7.53 | 10.49±6.50 | 0.020 | 14.87±6.92 | 15.16±7.97 | 0.925 | 13.88±5.90 | 10.48±6.44 | 0.060 | 15.42±4.20 | 13.28±6.45 | 0.427 |
| CD3+PD-1+(%) | 18.93±6.72 | 14.50±8.92 | 0.034 | 22.44±8.07 | 17.43±5.62 | 0.060 | 21.95±10.97 | 13.90±8.00 | 0.003 | 24.65±10.34 | 20.73±11.29 | 0.382 | 20.51±10.79 | 13.88±9.04 | 0.024 | 29.27±11.14 | 17.10±8.75 | 0.008 |
| PD-1+CD4+T(%) | 20.87±8.21 | 16.65±9.43 | 0.070 | 24.25±8.70 | 19.42±7.76 | 0.142 | 24.32±14.46 | 16.45±8.45 | 0.016 | 26.94±11.06 | 23.14±15.87 | 0.399 | 22.97±11.81 | 16.06±11.02 | 0.040 | 32.91±12.77 | 1911±9.11 | 0.006 |
| PD-1+CD8+T(%) | 15.35±6.72 | 13.74±10.61 | 0.452 | 16.05±7.58 | 15.05±6.50 | 0.717 | 18.15±9.84 | 12.77±9.49 | 0.018 | 20.49±9.75 | 17.09±9.94 | 0.599 | 18.11±9.86 | 12.24±7.24 | 0.009 | 21.76±10.45 | 16.69±9.54 | 0.256 |
| PD-1+CD4+CD8+T(%) | 39.10±18.37 | 30.67±21.27 | 0.960 | 44.57±21.47 | 37.51±19.04 | 0.377 | 40.51±26.00 | 25.39±22.97 | 0.049 | 47.06±27.00 | 35.36±26.35 | 0.282 | 38.27±22.71 | 33.23±21.19 | 0.601 | 47.96±27.92 | 32.28±21.92 | 0.150 |
| PD-1+CD4-CD8-T(%) | 19.19±10.78 | 9.88±1.79 | 0.004 | 24.10±10.12 | 14.67±8.36 | 0.013 | 20.84±12.87 | 11.19±13.03 | 0.029 | 26.76±13.50 | 15.32±10.34 | 0.018 | 21.46±12.92 | 7.40±9.41 | 0.001 | 30.86±16.74 | 15.09±8.51 | 0.048 |
| Treg PD-1+(%) | 27.72±14.28 | 21.11±12.94 | 0.065 | 34.02±16.21 | 25.03±12.84 | 0.115 | 30.76±16.07 | 18.95±9.75 | 0.002 | 39.48±15.63 | 26.84±15.02 | 0.048 | 28.76±18.04 | 19.55±12.90 | 0.045 | 43.61±20.04 | 22.98±13.84 | 0.007 |
| **CRP (mg/L)** | 153.83±74.80 | 79.88±62.91 | 1.12×10^-4^ | 156.09±84.12 | 152.86±72.66 | 0.916 | 135.66±77.37 | 106.21±77.56 | 0.177 | 124.85±86.89 | 140.21±75.08 | 0.647 | 116.36±89.18 | 37.04±244.48 | 0.145 | 148.93±128.82 | 105.51±73.35 | 0.710 |
| **PCT (ng/ml)** | 33.41±38.52 | 4.62±8.61 | 3.56×10^-4^ | 21.09±27.04 | 38.69±41.97 | 0.184 | 16.66±19.13 | 6.50±19.39 | 0.065 | 14.37±15.15 | 17.68±20.95 | 0.674 | 6.71±9.52 | 2.93±8.81 | 0.172 | 7.54±6.30 | 6.39±10.65 | 0.793 |
| **Lac (mmol/L)** | 3.00±3.60 | 1.86±1.35 | 0.109 | 4.26±5.23 | 2.44±2.55 | 0.214 | 1.97±1.44 | 1.47±1.05 | 0.248 | 2.44±1.28 | 1.72±1.49 | 0.233 | 1.57±0.92 | 1.52±0.73 | 0.863 | 2.24±1.12 | 1.18±0.50 | 0.046 |

S, Sepsis; NS, Non-sepsis; SD, Non-survivors with sepsis; SS, Survivors with sepsis. Neutrophil CD64 (nCD64) index was the ratio of the mean fluorescence intensity from neutrophils and lymphocytes. Monocyte CD64 (mCD64) index was the ratio of the mean fluorescence intensity from monocytes and lymphocytes.
